# Transcutaneous electrical nerve stimulation treated anterior talo-fibular ligament injured rat through the gut-joint axis and intestinal microbiota

**DOI:** 10.3389/fmicb.2026.1770614

**Published:** 2026-02-23

**Authors:** Nan Chen, Tong Ma, Ran Chen, Yang Zhang, Xun Tang, Yan Sun

**Affiliations:** 1 Trauma Center, First Affiliated Hospital of Kunming Medical University, Kunming Medical University, Kunming, Yunnan, China; 2 Key Laboratory of Pharmacology for Natural Products of Yunnan Province, Pharmaceutical College, Kunming Medical University, Kunming, Yunnan, China; 3 Clinical Lab, Second Affiliated Hospital of Kunming Medical University, Kunming, Yunnan, China; 4 CAS Key Laboratory of Animal Models and Human Disease Mechanisms, KIZ-SU Joint Laboratory of Animal Model and Drug Development, Laboratory of Learning and Memory, Kunming Institute of Zoology, The Chinese Academy of Sciences, Kunming, China

**Keywords:** ankle sprain, anterior talo-fibular ligament (ATFL), fecal microbiota transplantation (FMT), intestinal microbiota, transcutaneous electrical nerve stimulation (TENS)

## Abstract

**Objective:**

This study demonstrated that transcutaneous electrical nerve stimulation (TENS) and its induced fecal microbiota transplantation (FMT) could treat anterior talo-fibular ligament (ATFL) injury rat and modify the intestinal microbiota via the gut-joint axis.

**Methods:**

An ATFL injury model was duplicated and treated with low, medium, or high-intensity of TENS. After 1, 2, and 3 weeks of TENS treatment, the improvements and the expression levels of NOD2/IL-6/NF-κB/BMP-2/TGF-β were measured. The intestinal microbiota was analyzed via 16S rDNA sequencing. After FMT which induced by TENS, the improvement of ATFL injury rat was analyzed.

**Results:**

After TENS treatment, compared with the model control group, the bio-mechanical, gait, bone mineral density (BMD), etc. parameters were elevated in the TENS groups (*p* < 0.05); the expression of NOD2/IL-6 decreased and the BMP-2/TGF-β increased in the TENS groups (*p* < 0.05). The intestinal microbiota was altered, including increases in the abundances of *Erysipelotrichaceae*, *Lachnospira*, *Eubacterium*, *Phascolarctobacterium*, and *Alloprevotella*. After FMT, similar improvements were found in ATFL injury rats.

**Conclusion:**

TENS ameliorated ATFL injury rat by regulating the NOD2/IL-6/NF-κB/BMP-2/TGF-β and changed the intestinal microbiota through the gut-joint axis. Dominant intestinal microbiota was associated with FMT and could improve ATFL injury rat.

## 1 Introduction

The anterior talo-fibular ligament (ATFL) is one of the most important ligament structures of the ankle joint, and its main role is to maintain the stability of the ankle joint (Rougereau et al., 2024). Most ATFL injuries are caused by sports-related sprains and chronic ankle instability (CAI), which are manifested mainly as anterolateral ankle pain, joint instability and intermittent swelling (Mao et al., 2025). The treatment of ATFL injury mainly includes conservative treatment and surgical treatment. Conservative treatment means that first patients rest, walk with appropriate weight, gradually strengthen functional reconstruction, and finally strengthen the whole body to prevent recurrence (Sethi et al., 2023). However, conservative treatment has a long cycle and is difficult for patients to recover from persistent synovitis or tendinitis, ankle swelling, pain, and muscle weakness (Qin et al., 2025). At present, there are three widely used surgical treatments: the *Broström* method, the *Watson–Jones* method and ankle arthroscopy (Chen et al., 2023). Surgical treatment can produce adequate clinical results, but it does not result in adequate strength and is unable to satisfy the requirements of high-demand activities (Li et al., 2023).

Transcutaneous electrical nerve stimulation (TENS) is a non-injury-inducing treatment in which a specific low-frequency pulse current is applied into the human body through the skin to stimulate nerves for analgesia (Vance et al., 2022). TENS has advantages including safety, good analgesic effects, ability to reduce the use of opioids and capacity to circumvent the risk of infectious diseases induced by acupuncture (Szmit et al., 2023). TENS is a non-pharmacological intervention used to treat acute and chronic pain conditions in the clinic. There are two mechanisms of the analgesic effect of TENS: one relies on the gate control theory, and the other promotes the release of endogenous opioids, ultimately blocking the transmission of pain information to achieve pain relief (Chimenti et al., 2018).

The intestinal microbiota is composed of microorganisms and their genomic components and products in the gastrointestinal tract and has a relatively stable population structure (Altun et al., 2025). The microbiota is large in number and diverse. The human gut microbiota is composed of more than 100 trillion bacteria and more than 3 million unique genes, so the gut microbiota is called the “second genome” and “second brain” of the human body (Wang et al., 2023). An unhealthy imbalance in the composition of the microbial community, called “dysbiosis,” is closely associated with various metabolic, inflammatory, and intestinal barrier impairments. Research has suggested that *Bifidobacterium longum* CBi0703 administered orally over a period of 12 weeks decreased the number of cartilaginous lesions and decreased type II collagen degradation (Henrotin et al., 2021). Microbial DNA could be detected in intra-articular tissue and intra-cartilage tissue in the ankle joint injury (Zhao et al., 2018). Research has confirmed that some medicines have a positive effect on osteoarthritis by regulating the intestinal microbiota and serum metabolites (Jie et al., 2023). Recovery from injury to the ATFL is difficult, and patients can easily develop CAI and ankle osteoarthritis.

In this study, the rat models of ATFL damage were generated in duplicate and treated with TENS. After 1, 2, and 3 weeks of TENS treatment, the improvement of ATFL injury in rats was measured. Moreover, the osteogenic and anti-inflammatory effects of TENS in rats with ATFL injuries were analyzed. The intestinal microbiota of rats with ATFL injuries was subsequently analyzed via 16S rDNA sequencing. The ankle joint-gut axis was analyzed, and fecal microbiota transplantation (FMT) was performed using the dominant microbiota induced by TENS treatment in rats with ATFL injury. After 1, 2, or 3 weeks of FMT, the improvement, osteogenic and anti-inflammatory effects of FMT in rats with ATFL injuries were analyzed.

## 2 Materials and methods

### 2.1 TENS

TENS setting range as follows: the voltage peak value from 0.75–47.20 Vpp; the voltage values from 0.375–23.600 V; the current value 0.3675–23.1280 mA; the electric current density 0.1470–9.2512 A/m^2^; the frequency 25.00 Hz; stimulating the inner and outer sides of the knee joint in rats.

### 2.2 Animals

All animal experiments were approved by the Animal Study Committee of Kunming Medical University (No. KMMU2023MEC203) and were conducted according to the requirements of NIH Guidelines for care and use of laboratory animals. A total of 60 rats (Purchased from Kunming Medical University, Department of Animal, No. SYXK K2020-006), three-month-old SD female rats (200 ± 10 g) were maintained in standard conditions with a controlled temperature (21–23 °C) and a strict 12 h light/dark cycle. All the rats were fed with standard rat chow and allowed free access to distilled water *ad libitum* at all times during acclimatization and experimental treatment periods.

### 2.3 ATFL rat

Before the operation, fasting deprivation for 12 h, water deprivation for 6 h and the rats were anesthetized with intraperitoneal injection of sodium pentobarbital (0.1%, 35 mg/kg; Merck Ltd., Germany). ATFL model was constructed by partial resection of the anterior talo-fibular ligament of the right ankle. After the operation, ampicillin (6,000 IU/kg/day, HYZS Ltd., China) was injected intramuscularly for 3 days.

### 2.4 Grouping

A total of 36 ATFL rats were randomly divided into the model control (C) group, the low/medium/high (L/M/H) intensity of TENS group (low intensity: 3.16 Vpp, 1.58 V, 1.55 mA; medium intensity: 6.16 Vpp, 3.08 V, 3.02 mA; high intensity: 12.90 Vpp, 6.45 V, 6.32 mA) for 1/2/3 weeks.

Then a total of 24 ATFL rats were divided into eight groups, including the model control (C) group and the low/medium/high (L/M/H) intensity of TENS group treated with TENS (manufactured FMT which induced by TENS) or treated with FMT for 3 weeks.

### 2.5 Intestinal sample collection

When the animal model (the weights were 226.00–238.38 g, 249.25–255.00 g, 240.75–245.88 g after 1, 2, 3 weeks of induction) was euthanized by an overdose of anesthetic (150.0 mg/kg sodium pentobarbital, i.m.), the judgement standard of death was pupils dilated, absence of light reflex, breathing and heartbeat cease according to the Guideline for ethical review of animal welfare in Chinese. The intestinal samples were collected from the colon bags, including ATFL.1/2/3W.C.1/2/3, ATFL.1/2/3W.L/M/H.1/2/3. All samples are placed in sterile PBS and analyzed with 16S rDNA sequencing (Novogene Ltd., China).

### 2.6 16S rDNA sequencing

The intestinal samples were extracted by using the magnetic bead method of the Soil and Fecal Genomic cDNA Extraction Kit (TianGen, China) according to the manufacturer’s instructions. The V4–V5 region of the 16S rDNA gene was amplified by polymerase chain reaction (PCR) with a universal F′ and a unique bar-coded fusion R′ (341 F: ACTCCTACGGGAGGCAGCAG; 806 R: GGACTACHVGGGTWTCTAAT). Then, 15 mL of Phusion^®^ High-Fidelity PCR Master Mix (New England Biolabs), 0.2 mM primers and 10 ng of genomic DNA template was added to all PCR mixtures. The PCR conditions were as follows: denaturation at 98 °C for 1 min, followed by denaturation at 98 °C for 10 s, annealing at 50 °C for 30 s and extension at 72 °C for 30 s for a total of 30 cycles, and finally maintenance at 72 °C for 5 min. The amplicons were purified using AMPure beads (Axygen Co., United States). Barcoded libraries were generated by emulsion PCR and sequenced in the V4 to V5 reverse direction on a 318 chip using the 400 bp sequencing kit of the Ion Torrent Personal Genome Machine (PGM Co., United States) system according to the manufacturer’s instructions. The output sequences of each sample were no less than 50,000 pairs corresponding to 25,000 clean targets, and informatics methods (strategies: PE101/PE150/PE250/PE300, R language packages: QIIM2, ggplots) were applied.

### 2.7 Preparation of FMT

Briefly, 12 ATFL rats were randomly selected and divided into the four groups as previously and treated with TENS for 3 weeks as the donor rats. During the experimental cycle, the rats were induced to excrete fresh feces (3.0 g) by anal stimulation method at 9 a.m. of a day. Further sterile PBS was added, mixed and evenly dissolved (1:10), and vortex for about 0.5 min until there were no visible fecal particles. The collected samples were centrifuged at 2,000 rpm and 4 °C for 10 min and the fecal residue was discarded. Then the supernatant was centrifuged at 8,000 rpm, 4 °C for 5 min to obtain total bacteria. 0.1 mL of the diluted bacterial solution was cultured and calculated according to the formula viable count = average colony count × dilution ratio/dose volume. Finally, the 1 mL bacterial suspension (10^9^ CFU/mL) was transplanted to recipient rats one time per day for 3 weeks and the model control group was given 1 mL PBS. After the 3 weeks treatment with FMT, the rats were conducted with fasting deprivation for 12 h and the fecal samples were collected. The clearance of intestinal microbiota ahead of FMT with antibiotics metronidazole, neomycin C, ampicillin, vancomycin (50 mg: 50 mg: 50 mg: 25 mg diluted in 200 mL PBS, 200 μL/day per rat for 5 days).

### 2.8 Histopathological evaluation

The samples were harvested and fixed in 10% paraformaldehyde (Gefan Biotechnology Co., Ltd., China) for 14 days, dehydrated and gradually decalcified. Five-micrometre-thick sections were prepared using a Leica RM2245 microtome and stained with Hematein and Eosin (HE), Alcian blue (AB) & Alizarin red (Servicebio Co., China).

### 2.9 Evaluation of the *Mankin* score

The *Mankin* score quantifies the extent of cartilage degeneration by assessing the structure, cell number, matrix staining, and tideline integrity of the cartilage. A higher *Mankin* score indicates more severe cartilage degeneration as follows:

Cartilage structure: 0 for normal, 1 for surface irregularity, 2 for pannus formation and surface irregularity, 3 for fissure into the transition layer, 4 for fissure into the radiation layer, 5 for fissure into the calcification layer, and 6 for complete structural destruction.
Chondrocytes: 0 for normal, 1 for diffuse increased cells, 2 for focal increased cells, and 3 for significantly reduced cell number.
Cartilage matrix staining: 0 for normal, 1 for mild reduction, 2 for moderate reduction, 3 for severe reduction, and 4 for no staining.
Tideline integrity: 0 for completeness and 1 for vascular disruption.

### 2.10 Micro-computed tomography analysis

Micro-computed tomography (Micro-CT) analysis was performed according to recent guidelines^56^ using a SkyScan 1176 micro-CT imaging system (SkyScan, Bruker Ltd., Belgium) with a spatial resolution of 17.75 mm (X-ray source 70 kV/357 mA, 90 kV/270 mA; exposure time 250 ms/360 ms; magnification ×15; and 1.0 mm aluminum/0.1 mm copper filter). Volumetric reconstructions and analyses were performed using the built-in software NRecon 1.6 and CTAn 1.8. For the analysis of bone regeneration, the volume of interest was measured by the average grayscale value at the specific bone position (minimum to maximum degree: 0–255).

### 2.11 Immunohistological analysis

The sample were equilibrated in 0.1 Mtris-buffered saline for 10 min. After 1 hour of blockage in phosphate buffered saline (PBS) with 10% normal goat serum, the samples were incubated overnight at 4 °C with anti-bodies (NOD2, DF12125, 1:500, Affinity Co., China; IL-6, GB11117-50, 1:500, Servicebio Co., China). Then the samples were incubated with HRP-conjugated secondary antibody (GB23303, 1:500; Servicebio Co., China) for 1 h at room temperature. The results were enumerated by the ImageJ software (National Institutes of Health, Bethesda, MD, United States).

### 2.12 Step number of hind leg

The athletic ability of the ATFL rats was analyzed by calculating step number of hind leg which was recorded as the number of step number rats ran through a 25.00 cm exercise wheel. The rats were observed once a day for 6 days/week.

### 2.13 Bone biomechanics

A sample of the ankle was taken to keep the ligament-bone junction intact. Before starting the measurement, the length, width and thickness need to be measured (Muromachi Co., Japan), and then after bathing and stretching the fitting with normal saline, the ankle are fixed on the MTS universal materials testing machine to measure compression, bending and tensile related data.

### 2.14 AI gait analysis

Each rat was placed in a running wheel setup, where a camera recorded its movement at a frame rate of 30.00 Hz for a duration of 1 min. To ensure accurate tracking, key body parts were manually labeled in a set of key frames, including the knee (purple), ankle (cyan), and paw (red). The labeled dataset was used to train a deep learning model in DeepLabCut. Using a convolutional neural network, the model learned the spatial characteristics of each marker, enabling it to recognize and track these points in subsequent videos. Once trained, the model was applied to new videos for pose estimation and motion tracking, outputting coordinate data for each labeled body part. For analysis, the 1-min recording was divided into three 20-s segments. The following metrics were calculated for each segment:

Step length: Measured as the distance between successive placements of the paw marker.
Step frequency: Calculated based on the number of steps within each 20-s interval.
Ankle joint angle: Calculated as the angle formed between the knee and paw markers with the ankle as the vertex, providing insight into joint movement during running.

### 2.15 Virus tracing

A scAAV2/1-hSyn-EGFP-WPRE-pA virus (S0581-1, Taitool Bioscience Co. China) was injected into the joint cavity of ATFL rats, and the working concentration was 0.44 E13 VG (virus genome)/60 mL. After 3 weeks of treatment with TENS, the colon was collected and sliced at a thickness of 8 mm. Subsequently, the sections were analyzed with a microscope (Eclipse ci, NIS_F_Ver43000_64bit_E&Digital sight DS-FI2, NIKON Co., Japan).

### 2.16 Quantitative PCR

*IL-6* (Rattus), F′ CACTTCACAAGTCGGAGGCT, R’ AGCACACTAGGTTTGCCGAG; *NOD2* (Rattus), F′ GCAAGCACTTCCACTCCATC, R′ CAACTTGAGGTGCCCAACAT; *BMP-2* (Rattus), F′ GAAAACAGCAGCAGTGACC, R′ GGTGGCGTTCATGTAGGAGT; *TGF-β* (Rattus), F′ TGGGCACTGCTAGAGCCTAT, R′ GCGGAGATCCATACAAAGGA; *NF-κB* (Rattus), F′ TGTGAAGAAGCGAGACCTGG, R′ TGCTCCTCTATGGGAACTTGAA. *Erysipelotrichaceae*, F′ GGCGTGGATATGGTAGTGGT, R′ TAGTTCGAGCTCTGGTCTGC; *Lachnospira*, F′ TCATGCCTCCATTAGTTGTAAGCCT, R′ ATGAAGACTAATAACTCCAAAGAAAAAGTACGACAAC; *Eubacterium*, F′ ATGTTCAACGTAGGCGACCTGA, R′ TCAGTCGACGGTTCGGTCG; *Phascolarctobacterium*, F′ AACACATGCAAGTCGAACGG, R′ TTTCTTCATCCTGCCATGCG; *Alloprevotella*, F′ GTGAAAGTTCGGGGCTCAAC, R′ TCAGCGTCAGTTACACTCCG; 16S rRNA, F′ GTGCCAGCMGCCGCGGTAA, R′ TACCGCGGCTGCTGGCAC.

### 2.17 Western blot

The following antibodies were used: anti-GAPDH (GB15004, 1:5,000, Servicebio Co., China), anti-β-ACTIN (GB11001-100, 1:5,000, Servicebio Co., China), anti-BMP-2 (GB11252, 1:5,000, Servicebio Co., China), and anti-TGF-β1 (GB115750, 1:5,000, Servicebio Co., China), anti-NOD2 (DF12125, 1:5,000, Affinity Co., China), P65 (GB11997-100, 1:1,000, Servicebio Co., China).

### 2.18 Statistical analysis

Data was analyzed by GraphPad Prism 8 software (GraphPad, San Diego, CA, United States) and presented as the form of mean ± standard deviation (SD). The *p*-value of operational taxonomic unit (OTU) was performed by *MetagenomeSeq test*. The statistical differences were analyzed by *one-way analysis of* var*iance (ANOVA)* with *Tukey’s post-hoc test* for multiple group comparisons (SPSS 27.0, United States), and *p* < 0.05 indicated statistical significance.

## 3 Results

### 3.1 TENS treatment improves the function and bone quality in rats with ATFL injuries

**Figure 1:**
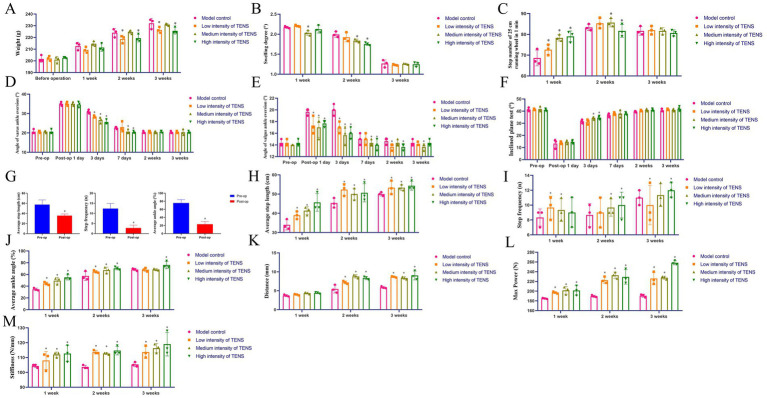
TENS treatment improves the function and bone quality in rats with ATFL injuries. **(A)** The weight of ATFL rats after 1, 2, 3 weeks of treatment with TENS. **(B)** The ankle swelling degree of ATFL rats after 1, 2, 3 weeks of treatment with TENS. **(C)** The step number of 25 cm in ATFL rat after 1, 2, 3 weeks of treatment with TENS. **(D,E)** The angle of varus and valgus ankle in ATFL rat after 1, 3 days and 1, 2, 3 weeks of treatment with TENS. **(F)** The degree of inclined plane test in ATFL rat after 1, 3 days and 1, 2, 3 weeks of treatment with TENS. **(G–J)** The average step length, step frequency, average ankle angle of ATFL rat which analyzed by AI gait analysis after 1, 2, 3 weeks of treatment with TENS. **(K–M)** The bio-mechanical distance, max power, stiffness in ATFL rat after 1, 2, 3 weeks of treatment with TENS. The values are presented as the mean ± standard deviation. ^*^p < 0.05 vs. the model control group, *n* = 3.

After 2 or 3 weeks of treatment with TENS, compared with those in the model control group, the weights in the low and high-intensity TENS groups decreased (*p* < 0.05, Figure 1A). After 2 weeks of treatment with TENS, compared with that in the model control group, the degree of swelling decreased in the medium and high-intensity TENS groups (*p* < 0.05, Figure 1B). After 1 week of treatment with TENS, the 25-cm step number increased in the TENS groups (*p* < 0.05, Figure 1C). After 3 days of treatment with TENS, the degree of varus and valgus ankle angles decreased in the TENS groups (*p* < 0.05, Figures 1D,E); the inclined plane test degrees increased in the medium and high-intensity TENS groups (*p* < 0.05, Figure 1F). Compared with those before the ATFL operation, the average step length, step frequency, and average ankle angle degree decreased postoperatively (*p* < 0.05, Figure 1G). After 3 weeks of treatment with TENS, compared with those in the model control group, the average step length and step frequency were greater in the TENS groups (*p* < 0.05, Figures 1H,I), and the average ankle angle was greater in the high-intensity TENS group (*p* < 0.05, Figure 1J). Compared with those in the model control group, the biomechanical distance, max power, and stiffiness were greater in the TENS groups (*p* < 0.05, Figures 1K–M).

### 3.2 Osteogenesis and anti-inflammatory effects of TENS in rats with ATFL injury via regulation of the NOD2/IF-6/NF-κB and BMP2/TGF-β signaling pathways

**Figure 2:**
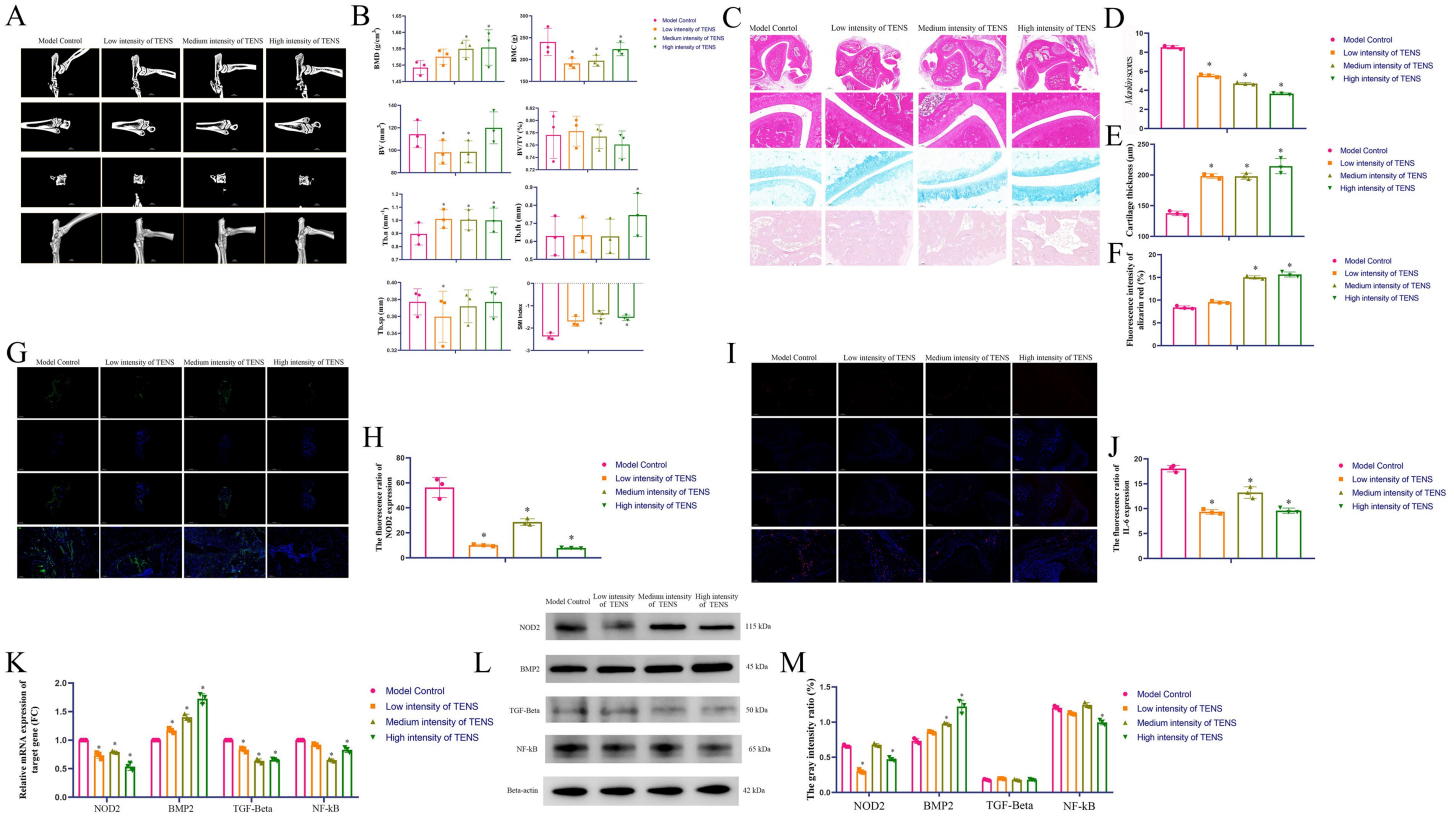
Osteogenesis and anti-inflammatory effects of TENS in rats with ATFL injury via regulation of the NOD2/IF-6/NF-κB and BMP2/TGF-β signaling pathways. **(A,B)** The micro-CT results after 3 weeks of treatment with TENS. **(C–F)** The evaluation of *Mankin* score, cartilage thickness, and the ratio of bone mass by HE, Alcian blue, Alizarin red staining, and ImageJ analysis after 3 weeks of treatment with TENS. The scale bar 100 μm. **(G,H)** The NOD2 expressions which analyzed by IF and ImageJ after 3 weeks of treatment with TENS. The NOD2 was green color and the scale bar 5000/100 μm. **(I,J)** The IL-6 expressions which analyzed by IF and ImageJ after 3 weeks of treatment with TENS. The IL-6 was red color and the scale bar 500/100 μm. **(K)** The NOD2/BMP2/TGF-β/NF-κB expressions which analyzed by qPCR after 3 weeks of treatment with TENS. The fold change (FC) was compared to the model control group (defined FC = 1). **(L,M)** The NOD2/BMP2/TGF-β/NF-κB expressions which analyzed by WB after 3 weeks of treatment with TENS. The index gray value vs. internal reference gray value represents the gray intensity ratio (%) which analyzed by ImageJ. The values are presented as the mean ± standard deviation. ^*^p < 0.05 vs. the model control group, *n* = 3.

Compared with that in the model control group, the bone mineral density (BMD) was greater in the medium and high-intensity TENS groups (*p* < 0.05, Figures 2A,B), the trabecular bone number (TB.n) value was greater in the TENS groups (*p* < 0.05), and the structure model index (SMI) was greater in the medium and high-intensity TENS groups (*p* < 0.05). Compared with those of the model control group, the *Mankin* scores were lower in the TENS groups (*p* < 0.05, Figures 2C,D), the cartilage thicknesses were greater in the TENS groups (*p* < 0.05, Figure 2E), and the bone mass ratios were greater in the medium and high-intensity TENS groups (*p* < 0.05, Figure 2F). Compared with that in the model control group, which was analyzed by immunofluorescence (IF), NOD2 expression was lower in the TENS groups (*p* < 0.05, Figures 2G,H), and IL-6 expression was lower in the TENS groups (*p* < 0.05, Figures 2I,J). After 3 weeks of treatment with TENS, compared with that in the model control group, NOD2 expression was lower in the TENS groups (*p* < 0.05, Figure 2K); BMP2 expression was greater in the TENS groups (*p* < 0.05); TGF-β expression was lower in the TENS groups (*p* < 0.05); and NF-κB expression was lower in the medium and high-intensity TENS groups (*p* < 0.05). Compared with that in the model control group, NOD2 expression was lower in the low and high-intensity TENS groups (*p* < 0.05, Figures 2L,M); BMP2 expression was greater in the medium and high-intensity TENS groups (*p* < 0.05); and NF-κB expression was lower in the high-intensity TENS group (*p* < 0.05).

### 3.3 Changes in the intestinal OTUs of rats with ATFL injury after TENS treatment

**Figure 3:**
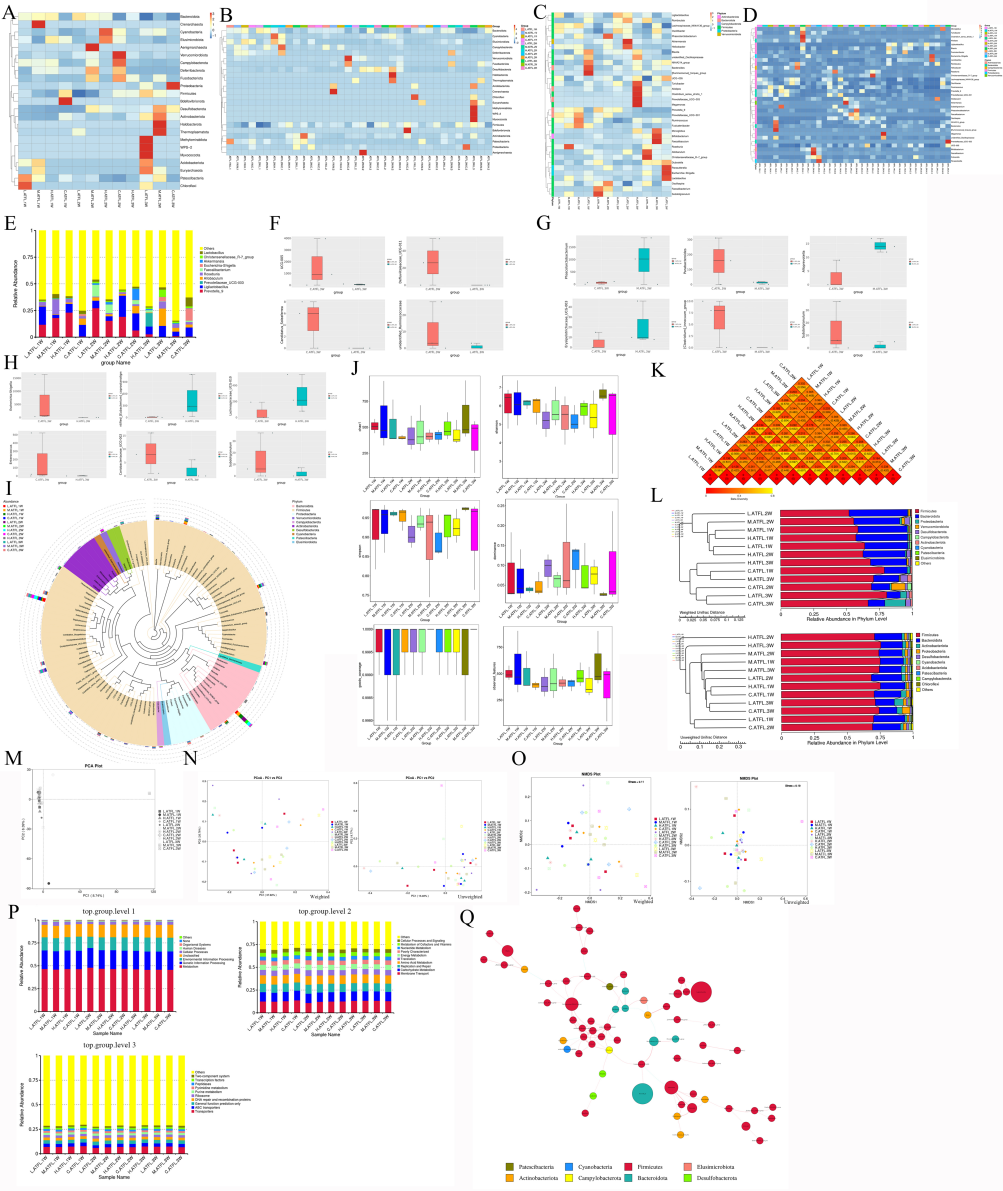
Changes in the intestinal OTUs of ATFL rats after TENS treatment. **(A)** The species abundance clustering plot of groups at phylum level. **(B)** The species abundance clustering plot of groups at genus level. The abscissa is the sample name. The ordinate represents the relative abundance. **(C–E)** The OTUs of ATFL rats after the treatment with L/M/H intensity of TENS. The ordinate is the absolute abundance of significantly divergent species. **(F)** Alpha diversity analysis of ATFL rats after TENS treatment. **(G,H)** The beta-diversities of all samples which analyzed by PCA, PCOA. The abscissa is one principal coordinate, the ordinate is the other principal coordinate. The closer the samples are, the more similar the species composition structure. Each point represented as a sample, and samples in the same group were represented as the same color. **(I)** The results of KO analysis level 1, 2, 3 which analyzed by PICRUST (Phylogenetic Investigation of Communities by Reconstruction of Unobserved States). The PICRUST functional analysis was based on KEGG database.

After 3 weeks of TENS treatment, the top 10 most abundant genera in rats with ATFL injury were selected (Figures 3A,B; Supplementary Figures 1A,B). After 3 weeks of TENS treatment, compared with the C.3W.ATFL group, the top 10 genera of the TENS group with increased OTUs included *Erysipelotrichaceae_UGG-003*, *Phascolarctobacterium*, *Alloprevotella*, *Eubacterium*, and *Lachnospiraceae_UCG-010*; moreover, the genera with decreased OTUs included *Candidatus_Soleaferrea*, *UCG-005*, *Rominococcacear*, etc. (Figure 3C–E; Supplementary Figure 1D). The top 11 representative sequences of the top 100 genera were obtained via multiple sequence alignment (Supplementary Figure 1C). Compared with those in the C.3W.ATFL group, the median, dispersion, maximum, and minimum values in the TENS groups were different in rats with ATFL injury after 3 weeks of TENS treatment (*p* < 0.05, Figure 3F). Compared with those in the C.3W.ATFL group, the unifrac distances were 0.164, 0.238, and 0.303, and the unweighted UniFrac distances were 0.374, 0.420, and 0.623 in the TENS groups (Supplementary Figures 1E,F). The separation rates of PC1 and PC2 were 8.74 and 6.39%, respectively, as determined via principal component analysis (PCA; Figure 3G). There were obvious differences between the model groups and the TENS groups, and the separation rates of PC1 and PC2 were 37.82%/13.48 and 20.70%/9.77%, respectively, as determined via principal coordinate analysis (PCoA) (Figure 3H). According to the non-metric multidimensional scaling (NMDS) analysis results, the stresses of the samples were greater than 0.02 (Supplementary Figure 1G).

After 3 weeks of treatment with TENS, compared with those of the control model group, the top 10 functions of all of the groups increased metabolism, genetic information processing, and environmental information processing (among other actions) at level 1 (Figure 3I); moreover, the top 10 functions of all of the groups increased membrane transport, carbohydrate metabolism, replication and repair (among other actions) at level 2. The top 10 functions of all of the groups increased transporters, ABC transporters, general function prediction only (among other actions) (Shi et al., 2023). The microbiota exhibiting changed abundances after treatment with TENS were related to each other, and the predicted functions worked in a cosynergistic manner (Supplementary Figure 1H).

### 3.4 FMT which induce by TENS improves ankle function and bone quality via the gut-knee joint axis in ATFL rats

**Figure 4:**
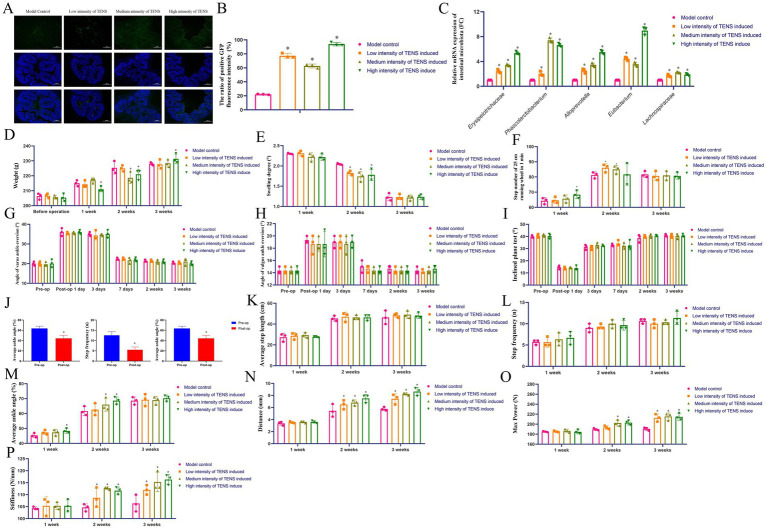
FMT which induce by TENS improves ankle function and bone quality via the gut-knee joint axis in rats with ATFL injuries. **(A,B)** The virus tracing of gut in ATFL rat was stained by IF after 3 weeks of virus tracing. Positive GFP label was color green. **(C)** The intestinal microbiota expressions of FMT was analyzed by qPCR. The fold change (FC) was compared to the 16S rRNA (defined FC = 1). **(D)** The weight of ATFL rats after 1, 2, 3 weeks of treatment with FMT. **(E)** The ankle swelling degree of ATFL rats after 1, 2, 3 weeks of treatment with FMT. **(F)** The step number of 25 cm in ATFL rat after 1, 2, 3 weeks of treatment with FMT. **(G,H)** The angle of varus and valgus ankle in ATFL rat after 1, 3 days and 1, 2, 3 weeks of treatment with FMT. **(I)** The degree of inclined plane test in ATFL rat after 1, 3 days and 1, 2, 3 weeks of treatment with FMT. **(J–M)** The average step length, step frequency, average ankle angle of ATFL rat which analyzed by AI gait analysis after 1, 2, 3 weeks of treatment with FMT. **(N–P)** The bio-mechanical distance, max power, stiffness in ATFL rat after 1, 2, 3 weeks of treatment with FMT. The values are presented as the mean ± standard deviation. ^*^p < 0.05 vs. the model control group, *n* = 3.

After 3 weeks of treatment with TENS, the positive GFP fluorescence intensity of virus tracing increased in the TENS groups (*p* < 0.05, Figures 4A,B); The FMT which induced by 3 weeks of treatment with TENS was manufactured for ATFL rats and the dominant intestinal microbiota was analyzed by qPCR as the result of 16S rDNA analysis (*p* < 0.05, Figure 4C). After 2 weeks of treatment with FMT, the weights in the medium/high intensity of TENS groups decreased compared to those of the model control group (*p* < 0.05, Figure 4D). After 2 weeks of treatment with FMT, compared with that in the model control group, the ankle swelling degrees decreased in the TENS groups (*p* < 0.05, Figure 4E). After 2 weeks of treatment with FMT, the step number of 25 cm increased in the low/medium intensity of TENS groups (*p* < 0.05, Figure 4F). After treatment with FMT, the angle of varus and valgus ankle degree, the degree of inclined plane test was similar (*p* > 0.05, Figures 4G–I). After ATFL operation, compared with the pre-op, the average step length, step frequency, degree of average ankle angle decreased in the post-op (*p* < 0.05, Figure 4J). After treatment with FMT, compared with that in the model control group, the average step length and step frequency was similar (*p* > 0.05, Figures 4K,L); after 2 weeks of treatment with FMT, the average ankle angle increased in the medium/high TENS groups (*p* < 0.05, Figure 4M). After 3 weeks of treatment with FMT, compared with that in the model control group, the bio-mechanical distance, max power, stiffiness increased in the TENS groups (*p* < 0.05, Figures 4N–P).

### 3.5 FMT induced by TENS improved ATFL injury in rats by regulating the NOD2/BMP2/TGF-β signaling pathway

**Figure 5:**
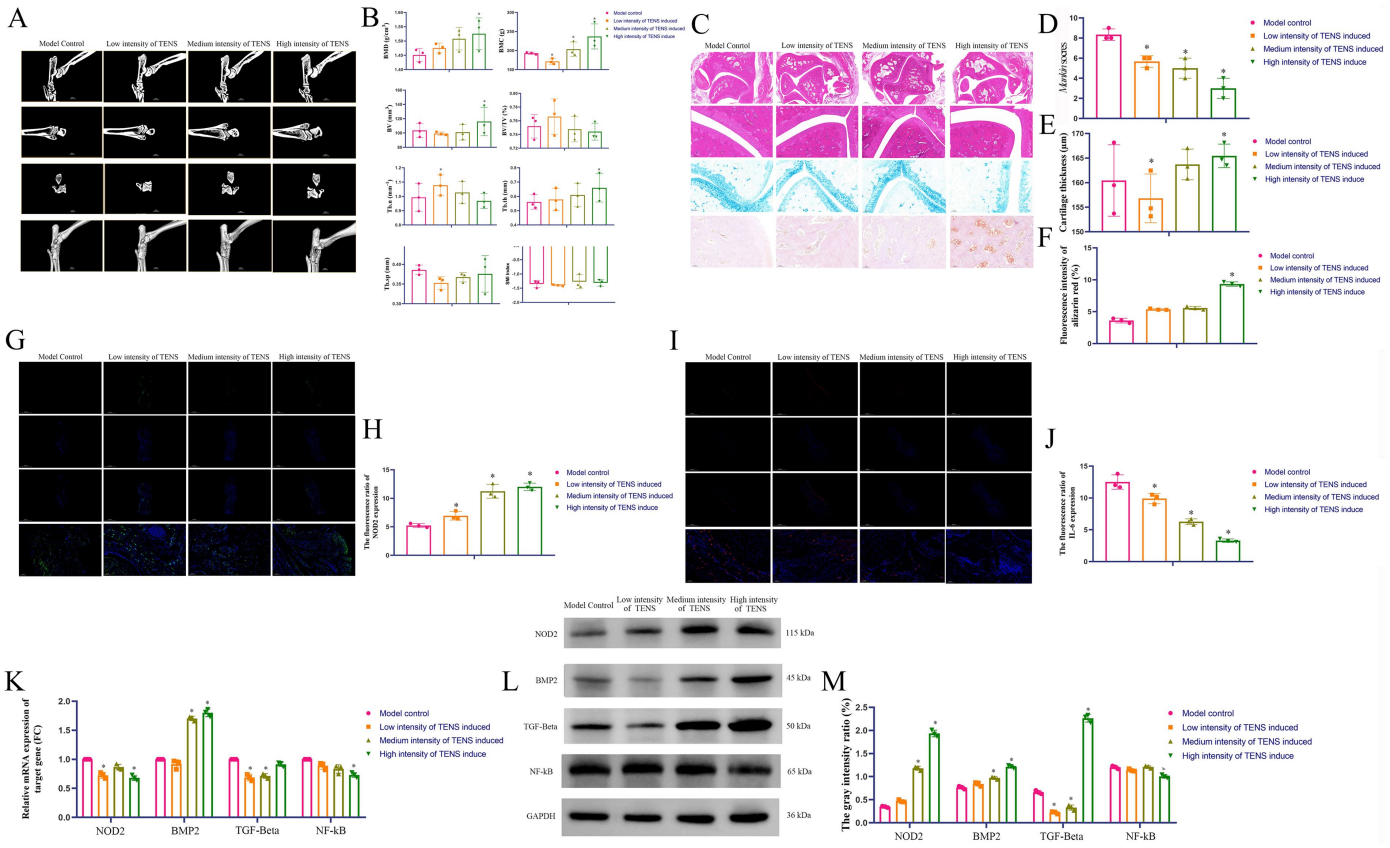
FMT induced by TENS improved ATFL injury in rats by regulating the NOD2/BMP2/TGF-β signaling pathway. **(A,B)** The micro-CT results after 3 weeks of treatment with FMT. **(C–F)** The evaluation of *Mankin* score, cartilage thickness, and the ratio of bone mass by HE, Alcian blue, Alizarin red staining, and ImageJ analysis after 3 weeks of treatment with TENS. The scale bar 100 μm. **(G,H)** The NOD2 expressions which analyzed by IF and ImageJ after 3 weeks of treatment with TENS. The NOD2 was green color, and the scale bar 5,000/100 μm. **(I,J)** The IL-6 expressions which analyzed by IF and ImageJ after 3 weeks of treatment with FMT. The IL-6 was red color, and the scale bar 5,000/100 μm. **(K)** The NOD2/BMP2/TGF-β/NF-κB expressions which analyzed by qPCR after 3 weeks of treatment with FMT. The fold change (FC) was compared to the model control group (defined FC = 1). **(L,M)** The NOD2/BMP2/TGF-β/NF-κB expressions which analyzed by WB after 3 weeks of treatment with FMT. The index gray value vs. internal reference gray value represents the gray intensity ratio (%) which analyzed by ImageJ. The values are presented as the mean ± standard deviation. ^*^p < 0.05 vs. the model control group, *n* = 3.

Compared with those in the model control group, the BMD, bone mineral content (BMC), TB.n and trabecular bone thickness (TB.th) in the high-intensity TENS group increased after 3 weeks of treatment with FMT (*p* < 0.05, Figures 5A,B). Compared with those in the model control group, the *Mankin* scores were lower in the TENS groups (*p* < 0.05, Figures 5C,D), and the cartilage thickness and the ratio of bone mass were greater in the high-intensity TENS group (*p* < 0.05, Figures 5E,F). Compared with that in the model control group, NOD2 expression was higher in the TENS groups (*p* < 0.05, Figures 5G,H), and IL-6 expression was lower in the TENS groups (*p* < 0.05, Figures 5I,J). After 3 weeks of treatment with FMT, compared with that in the model control group, NOD2 expression was lower in the low and high-intensity TENS groups (*p* < 0.05, Figure 5K); BMP2 expression was greater in the medium and high-intensity TENS groups (*p* < 0.05); TGF-β expression was lower in the low and medium-intensity TENS groups (*p* < 0.05); and NF-κB expression was lower in the high-intensity TENS group (*p* < 0.05). After 3 weeks of FMT, compared with those in the model control group, NOD2 and BMP2 expression increased in the medium and high-intensity TENS groups (*p* < 0.05, Figures 5L,M); TGF-β expression decreased in the low and medium-intensity TENS groups (*p* < 0.05); and NF-κB expression decreased in the high-intensity TENS group (*p* < 0.05).

## 4 Discussion

This study revealed the improvement of ATFL injury in rats via TENS, and its mechanism was related to osteogenesis and anti-inflammatory effects on the basis of surgery that is considered the gold standard for ATFL injury treatment. After TENS treatment, the body weights in the low and high-intensity TENS group decreased, and the body weights were better for surgical management (Miranda et al., 2024); the degree of ankle swelling decreased in the TENS groups, indicating that TENS could at least improve swelling in rats with ATFL injury. As mentioned above, TENS is analgesic and anti-inflammatory after surgery (Lee et al., 2024). The rats with ATFL injury in the TENS groups presented better motor ability in this study. An AI gait method of analyses motor ability in terms of step length, frequency and ankle degree. Some automated methods, such as gait analysis and deep learning, have been used in the diagnosis of ATFL injury (Ni et al., 2023). Moreover, the biomechanical indices of rats with ATFL injury improved after TENS treatment. A study revealed that quantitatively evaluating the biomechanical effects of ATFL and CFL lesions could aid in the repair of ATFL injury, and a better biomechanical structure improved the stability of the ankle (Larkins et al., 2021). Moreover, the results of this study revealed better microstructures in the TENS groups than in the model control group. TENS increased the micro-CT index, cartilage thickness, and bone mass and decreased the *Mankin* score in rats with ATFL injury. A simple validation experiment revealed the anti-inflammatory effects of TENS in rats with ATFL injury via the downregulation of IF-6/NF-κB expression, as described in previous research. The improvement in bone quality in the TENS groups was related to the regulation of NOD2/BMP2/TGF-β signaling. The experimental design was flawed, and we cannot explain why NOD2/BMP2/TGF-β signaling was dysregulated after TENS treatment. A related-omics study by our group revealed the NOD2/BMP2/TGF-β signaling pathway. Decreasing the expression of NOD2 could increase ALP secretion by primary osteoblasts in high-glucose medium (Chen et al., 2024). NOD2, a member of the NOD-like receptor family of PRRs, is an important mediator of ER stress-induced inflammation in mouse and human cells. The ER stress inducers thapsigargin and dithiothreitol trigger the production of the proinflammatory cytokine IL-6 in a NOD1/2-dependent fashion via the NF-κB pathway (Keestra-Gounder et al., 2016). BMP2, a pluripotent factor, is a member of the TGF-β super-family. It is a classic BMP2/TGF-β signaling pathway whose function is related to bone formation and an increase in the number of articular chondrocytes (Yang et al., 2024). A study revealed that NOD2 expression deficiency promoted cardiac hypertrophy and fibrosis; it enhanced the activation of the NF-κB and TGF-β/Smad pathways in NOD2-knockout mice which compared with WT mice (Zong et al., 2013). In summary, TENS activated NOD2 to ablate the anti-inflammatory effects of IF-6/NF-κB; meanwhile, TENS activated the BMP2/TGF-β pathway. An interesting finding related to ATFL and FMT was presented. After 3 weeks of FMT, NOD2 protein expression, as well as BMP2 protein expression, increased in the medium and high-intensity TENS groups. We speculate that TENS could improve ATFL injury through the regulation of the NOD2/IF-6/NF-κB/BMP2/TGF-β signaling pathway. However, the expression of NOD2 increased in the TENS group and NF-κB decreased in the high intensity of TENS group after FMT. FMT induced by TENS could affect the regulation of ATFL injury rats via multiple repetitions and the exclusion of interfering factors.

The intestinal microbiota of rats with ATFL injury was analyzed via 16S rDNA sequencing after 1, 2, or 3 weeks of treatment with TENS. In this study, we performed a sequential analysis of the changes in the intestinal microbiota, and these changes were not accidental. Several dominant genera affected the improvement of ATFL injury. First, the ankle joint-gut axis was confirmed in this study. The intestinal microbiota produces a variety of compounds that move from the “leaky gut” to the bloodstream, thereby leading to joint disease (Longo et al., 2024). ATFL injury is closely related to the imbalance of the intestinal microbiota, and the ATFL injuries in rats were artificially induced in this study. Five dominant genera were *Erysipelotrichaceae*, *Alloprevotella*, *Eubacterium*, *Lachnospiraceae*, and *Phascolarctobacterium* in rats with ATFL injury after 3 weeks of treatment with TENS. *Erysipelotrichaceae* might affect intestinal diseases, lung cancer, depression, etc. A study found that the change of *Erysipelotrichaceae* could affect the systemic immunity and inflammatory responses in a amyotrophic lateral sclerosis rats model; its regulation was related to the metabolites especially the levels of short and medium-chain fatty acids (Niccolai et al., 2024). NOD2 is an intracellular PRR that senses bacterial peptidoglycan in the cytosol, and *Erysipelotrichaceae* increases the risk of inflammatory bowel disease (Turpin et al., 2020). If a oral bacteria infection duplicated in the C57Bl/6 mouse, the alveolar bone loss was been found and the changed intestinal micriobiota were *Erysipelotrichaceae*, *Alloprevotella*, *Rothia* (Rocha et al., 2024). *Alloprevotella* could become an oral biomarker to diagnose the intestinal metaphase phase of gastric patients, and it improved gut microbiota dysbiosis in obese mice fed a high-fat or high-sucrose diet (Liu et al., 2023). *Alloprevotella* exhibited a positive correlation with the baseline level of serum phosphorus in patients who suffered from bone loss (Coskun et al., 2024). Many studies have focused on energy homeostasis, colonic motility, immunomodulation and the suppression of inflammation by *Eubacterium* (Shi et al., 2023). *Lachnospiraceae* belong to the core of the gut microbiota and are among the main producers of short-chain fatty acids (Vacca et al., 2020). *Phascolarctobacterium* can produce short-chain fatty acids, and a gradual increase in the number of bacteria is maintained at a high level with increasing age (Ciobârcă et al., 2025). Studies have shown that *Phascolarctobacterium* is related to neurological and psychiatric diseases (Zang et al., 2023). In this study, these intestinal microbiota and their metabolic components, including genetic information processing, environmental information processing, and membrane transport, participated in the progression of ATFL injury. Currently, it exists no direct evidence to clarify the mechanism through which the microbiota facilitates osteogenesis and exerts anti-inflammatory effects; now, we did the research to reveal relationship between the intestinal micirobiota, metabolites, bone disease and it could bring a new perspectives for treating the KOA. Although osteogenesis was activated subsequent to TENS, it is hypothesized that the ligament condition of ATFL injury in rats was not repaired; and the improvement was contingent upon anti-inflammatory effects, enhanced local bone microstructure, and increased cartilage thickness, among other factors. A research report indicated that the *Lactiplantibacillus* plantarum EIR/IF-1 strain demonstrated a regulatory effects on the inflammatory response to lipopolysaccharide stimulation, cell migration, cell proliferation, and collagen synthesis in human periodontal ligament mesenchymal stromal cells (Demirhan et al., 2025).

To verify the effects of the dominant genera on improving ATFL injury in rats, fecal microbiota transplantation (FMT), which was induced by different stimulated TENS, was used. FMT involves the transfer of stool from a healthy donor into the colon of a patient with the goal of restoring the normal microbiota and thus curing the disease (Logoń et al., 2023). In this study we manufactured four FMTs (one from control rats with ATFL injury; three were from rats subjected to different intensities of TENS) and treated rats with ATFL injuries for 3 weeks. Similar results were obtained, and FMT induced by TENS improved ATFL injury. However, improvements in the results of the behavioural analysis were not apparent over a short period of treatment. A study of FMT and obesity lasted for 12 weeks, and its treatment took into account the clinical disease (Sivalingam et al., 2021). The experimental terminus was set 3 weeks after TENS or FMT because ATFL injury patients who did not undergo surgery showed significant improvement in inflammation after 3 weeks. The improvements related to FMT may have occurred later than those related to TENS, and FMT is a long-term treatment. The regulation of NOD2/IF-6/NF-κB and BMP2/TGF-β could provide insight into the mechanism of TENS and FMT for ATFL injury. TENS can regulate these gene targets, and its induced dominant genus also regulates these gene targets. Although TENS and FMT could improve ATFL injury via the ankle joint-gut axis, we still do not know that certain FMT compounds target NOD2 expression and promote osteogenesis and anti-inflammatory effects. The regulation of FMT is complicated, and it is necessary to analyse the contents of the intestinal microbiota in future studies.

## 5 Conclusion

TENS improved ATFL injury in rats, and its mechanism of osteogenic and anti-inflammatory effects was related to the regulation of the NOD2/IF-6/NF-κB and BMP2/TGF-β signaling pathways. TENS can change the intestinal microbiota via the ankle joint-gut axis, and FMT induced by TENS can also improve ATFL injury in rats. TENS, which can induce FMT, even compounds the dominant genus, has the potential to treat ATFL injury in the clinic.

## Data Availability

The datasets presented in this study can be found in online repositories. The names of the repository/repositories and accession number(s) can be found at: 10.17632/nbzb98ks3x.1.

## References

[ref1] Altun M. Küçük U. Yıldırım N. (2025). Modulation of gut microbiota using VSL#3 and its impact on aortic parameters in a rat model. Anatol. J. Cardiol. 29, 282–290. doi: 10.14744/AnatolJCardiol.2025.5048, 40114628 PMC12151109

[ref2] Chen Y. Y. Tan L. Su X. L. Chen N. X. Liu Q. Feng Y. Z. . (2024). NOD2 contributes to *Parvimonas micra*-induced bone resorption in diabetic rats with experimental periodontitis. Mol. Oral Microbiol. 39, 446–460. doi: 10.1111/omi.12467, 38757737

[ref3] Chen R. P. Wang Q. H. Li M. Y. Su X. F. Wang D. Y. Liu X. H. . (2023). Progress in diagnosis and treatment of acute injury to the anterior talofibular ligament. World J. Clin. Cases 11, 3395–3407. doi: 10.12998/wjcc.v11.i15.3395, 37383912 PMC10294195

[ref4] Chimenti R. L. Frey-Law L. A. Sluka K. A. (2018). A mechanism-based approach to physical therapist management of pain. Phys. Ther. 98, 302–314. doi: 10.1093/ptj/pzy030, 29669091 PMC6256939

[ref5] Ciobârcă D. Cătoi A. F. Gavrilaș L. Banc R. Miere D. Filip L. (2025). Natural bioactive compounds in the management of type 2 diabetes and metabolic (dysfunction)-associated steatotic liver disease. Pharmaceuticals 18:279. doi: 10.3390/ph18020279, 40006091 PMC11859434

[ref6] Coskun M. Babayeva A. Barlas T. Muhittin Yalcin M. Akturk M. Balos Toruner F. . (2024). Relationship between gut microbiome and bone deficits in primary hyperparathyroidism: a proof-of-concept pilot study. J. Investig. Med. 72, 541–552. doi: 10.1177/10815589241251695, 38641855

[ref7] Demirhan H. K. Omer Oglou E. Aksoy Z. B. Kiran F. (2025). Evaluation of the anti-inflammatory, antioxidant and regenerative effects of microbiota-derived postbiotics in human periodontal ligament mesenchymal stromal cells. Clin. Oral Investig. 29:262. doi: 10.1007/s00784-025-06341-1, 40263129 PMC12014813

[ref8] Henrotin Y. Patrier S. Pralus A. Roche M. Nivoliez A. (2021). Protective actions of oral administration of *Bifidobacterium longum* CBi0703 in spontaneous osteoarthritis in Dunkin Hartley guinea pig model. Cartilage 13, 1204S–1213S. doi: 10.1177/1947603519841674, 30982336 PMC8804838

[ref9] Jie L. Ma Z. Gao Y. Shi X. Yu L. Mao J. . (2023). The mechanism of palmatine-mediated intestinal flora and host metabolism intervention in OA-OP comorbidity rats. Front. Med. 10:1153360. doi: 10.3389/fmed.2023.1153360, 37153081 PMC10159182

[ref10] Keestra-Gounder A. M. Byndloss M. X. Seyffert N. Young B. M. Chávez-Arroyo A. Tsai A. Y. . (2016). NOD1 and NOD2 signaling links ER stress with inflammation. Nature 532, 394–397. doi: 10.1038/nature17631, 27007849 PMC4869892

[ref11] Larkins C. G. Brady A. W. Aman Z. S. Dornan G. J. Haytmanek C. T. Clanton T. O. (2021). Evaluation of the intact anterior talofibular and calcaneofibular ligaments, injuries, and repairs with and without augmentation: a biomechanical robotic study. Am. J. Sports Med. 49, 2432–2438. doi: 10.1177/03635465211018645, 34110933

[ref12] Lee T. Y. Chen P. Y. Yang K. C. Tzeng I. S. Ming Chang C. Wang C. C. (2024). Comparison of knot-tying techniques during the arthroscopic Broström-Gould procedure: semiconstrained freehand versus knot pusher techniques. Orthop. J. Sports Med. 12:23259671231218649. doi: 10.1177/23259671231218649, 38274016 PMC10809873

[ref13] Li Q. Fu X. Kou Y. Han N. (2023). Engineering strategies and optimized delivery of exosomes for theranostic application in nerve tissue. Theranostics 13, 4266–4286. doi: 10.7150/thno.84971, 37554270 PMC10405842

[ref14] Liu Y. Wang H. Jiang H. Sun Z. Sun A. (2023). Alloprevotella can be considered as a potential oral biomarker in intestinal metaphase of gastric patients. Stud. Health Technol. Inform. 308, 155–167. doi: 10.3233/SHTI230836, 38007737

[ref15] Logoń K. Świrkosz G. Nowak M. Wrześniewska M. Szczygieł A. Gomułka K. (2023). The role of the microbiome in the pathogenesis and treatment of asthma. Biomedicine 11:1618. doi: 10.3390/biomedicines11061618, 37371713 PMC10295573

[ref16] Longo U. G. Lalli A. Bandini B. de Sire R. angeletti S. Lustig S. . (2024). Role of the gut microbiota in osteoarthritis, rheumatoid arthritis, and spondylarthritis: an update on the gut-joint axis. Int. J. Mol. Sci. 25:3242. doi: 10.3390/ijms25063242, 38542216 PMC10970477

[ref17] Mao W. Jin Z. Li W. Zhu Y. Kong W. Wang Y. . (2025). All-inside arthroscopic repair of ATFL and CFL separately for chronic lateral ankle instability in conjunction with subtalar instability. J. Orthop. Surg. Res. 20:380. doi: 10.1186/s13018-025-05780-6, 40234894 PMC12001407

[ref18] Miranda S. Harahap A. Husada D. Faramarisa F. N. (2024). Risk factors of multidrug-resistant organisms neonatal sepsis in Surabaya tertiary referral hospital: a single-center study. BMC Pediatr. 24:153. doi: 10.1186/s12887-024-04639-9, 38424519 PMC10902940

[ref19] Ni M. Chen W. Zhao Q. Zhao Y. Yuan H. (2023). Deep learning approach for MRI in the classification of anterior talofibular ligament injuries. J. Magn. Reson. Imaging 58, 1544–1556. doi: 10.1002/jmri.28649, 36807381

[ref20] Niccolai E. Di Gloria L. Trolese M. C. Fabbrizio P. Baldi S. Nannini G. . (2024). Host genetics and gut microbiota influence lipid metabolism and inflammation: potential implications for ALS pathophysiology in SOD1G93A mice. Acta Neuropathol. Commun. 12:174. doi: 10.1186/s40478-024-01877-x, 39506789 PMC11539544

[ref21] Qin Q. N. Zhang X. Cai Y. F. Tian T. Z. Zhou J. P. Li L. . (2025). Heel kicking exercise rapidly improves pain and function in patients with acute lateral ankle sprain: a randomized controlled trial. BMC Musculoskelet. Disord. 26:666. doi: 10.1186/s12891-025-08881-9, 40629319 PMC12235833

[ref22] Rocha C. M. Kawamoto D. Martins F. H. Bueno M. R. Ishikawa K. H. Ando-Suguimoto E. S. . (2024). Experimental inoculation of *Aggregatibacter actinomycetemcomitans* and *Streptococcus gordonii* and its impact on alveolar bone loss and oral and gut microbiomes. Int. J. Mol. Sci. 25:8090. doi: 10.3390/ijms25158090, 39125663 PMC11312116

[ref23] Rougereau G. Marty-Diloy T. Vigan M. Donadieu K. Vialle R. Langlais T. . (2024). Biomechanical evaluation of the anterior talo-fibular and calcaneo-fibular ligaments using shear wave elastography in young healthy adults. Orthop. Traumatol. Surg. Res. 110:103647. doi: 10.1016/j.otsr.2023.103647 37356798

[ref24] Sethi M. Limaye R. Rai A. Limaye N. (2023). Anterior talo-fibular ligament reconstruction with InternalBrace^™^ for chronic lateral ankle instability in Pediatric patients. Cureus 15:e44979. doi: 10.7759/cureus.44979, 37822443 PMC10563824

[ref25] Shi R. Yu F. Hu X. Liu Y. Jin Y. Ren H. . (2023). Protective effect of *Lactiplantibacillus plantarum* subsp. *plantarum* SC-5 on dextran sulfate sodium-induced colitis in mice. Foods 12:897. doi: 10.3390/foods12040897, 36832972 PMC9957050

[ref26] Sivalingam S. Larsen E. L. van Raalte D. H. Muskiet M. H. A. Smits M. M. Tonneijck L. . (2021). The effect of liraglutide and sitagliptin on oxidative stress in persons with type 2 diabetes. Sci. Rep. 11:10624. doi: 10.1038/s41598-021-90191-w, 34012064 PMC8134438

[ref27] Szmit M. Krajewski R. Rudnicki J. Agrawal S. (2023). Application and efficacy of transcutaneous electrical acupoint stimulation (TEAS) in clinical practice: a systematic review. Adv. Clin. Exp. Med. 32, 1063–1074. doi: 10.17219/acem/159703, 37026972

[ref28] Turpin W. Bedrani L. Espin-Garcia O. Xu W. Silverberg M. S. Smith M. I. . (2020). Associations of NOD2 polymorphisms with Erysipelotrichaceae in stool of in healthy first degree relatives of Crohn's disease subjects. BMC Med. Genet. 21:204. doi: 10.1186/s12881-020-01115-w, 33059653 PMC7566148

[ref29] Vacca M. Celano G. Calabrese F. M. Portincasa P. Gobbetti M. De angelis M. (2020). The controversial role of human gut Lachnospiraceae. Microorganisms 8:573. doi: 10.3390/microorganisms8040573, 32326636 PMC7232163

[ref30] Vance C. G. T. Dailey D. L. Chimenti R. L. Van Gorp B. J. Crofford L. J. Sluka K. A. (2022). Using TENS for pain control: update on the state of the evidence. Medicina 58:1332. doi: 10.3390/medicina58101332, 36295493 PMC9611192

[ref31] Wang Y. H. Yan Z. Z. Luo S. D. Hu J. J. Wu M. Zhao J. . (2023). Gut microbiota-derived succinate aggravates acute lung injury after intestinal ischaemia/reperfusion in mice. Eur. Respir. J. 61:2200840. doi: 10.1183/13993003.00840-2022, 36229053

[ref32] Yang D. H. Nah H. Lee D. Min S. J. Park S. An S. H. . (2024). A review on gold nanoparticles as an innovative therapeutic cue in bone tissue engineering: prospects and future clinical applications. Mater. Today Bio 26:101016. doi: 10.1016/j.mtbio.2024.101016, 38516171 PMC10952045

[ref33] Zang Y. Lai X. Li C. Ding D. Wang Y. Zhu Y. (2023). The role of gut microbiota in various neurological and psychiatric disorders-an evidence mapping based on quantified evidence. Mediat. Inflamm. 2023:5127157. doi: 10.1155/2023/5127157, 36816743 PMC9936509

[ref34] Zhao Y. Chen B. Li S. Yang L. Zhu D. Wang Y. . (2018). Detection and characterization of bacterial nucleic acids in culture-negative synovial tissue and fluid samples from rheumatoid arthritis or osteoarthritis patients. Sci. Rep. 8:14305. doi: 10.1038/s41598-018-32675-w, 30250232 PMC6155189

[ref35] Zong J. Salim M. Zhou H. Bian Z. Y. Dai J. Yuan Y. . (2013). NOD2 deletion promotes cardiac hypertrophy and fibrosis induced by pressure overload. Lab. Investig. 93, 1128–1136. doi: 10.1038/labinvest.2013.99, 23958879

