# Supplementary material for: Transcutaneous electrical nerve stimulation treated anterior talo-fibular ligament injured rat through the gut-joint axis and intestinal microbiota
Source: Front Microbiol. 2026 Feb 23;17:1770614. doi: 10.3389/fmicb.2026.1770614 (PMC12968229; doi:10.3389/fmicb.2026.1770614)
Supplement: Supplementary file 1 [file Supplementary_file_1.docx]

**
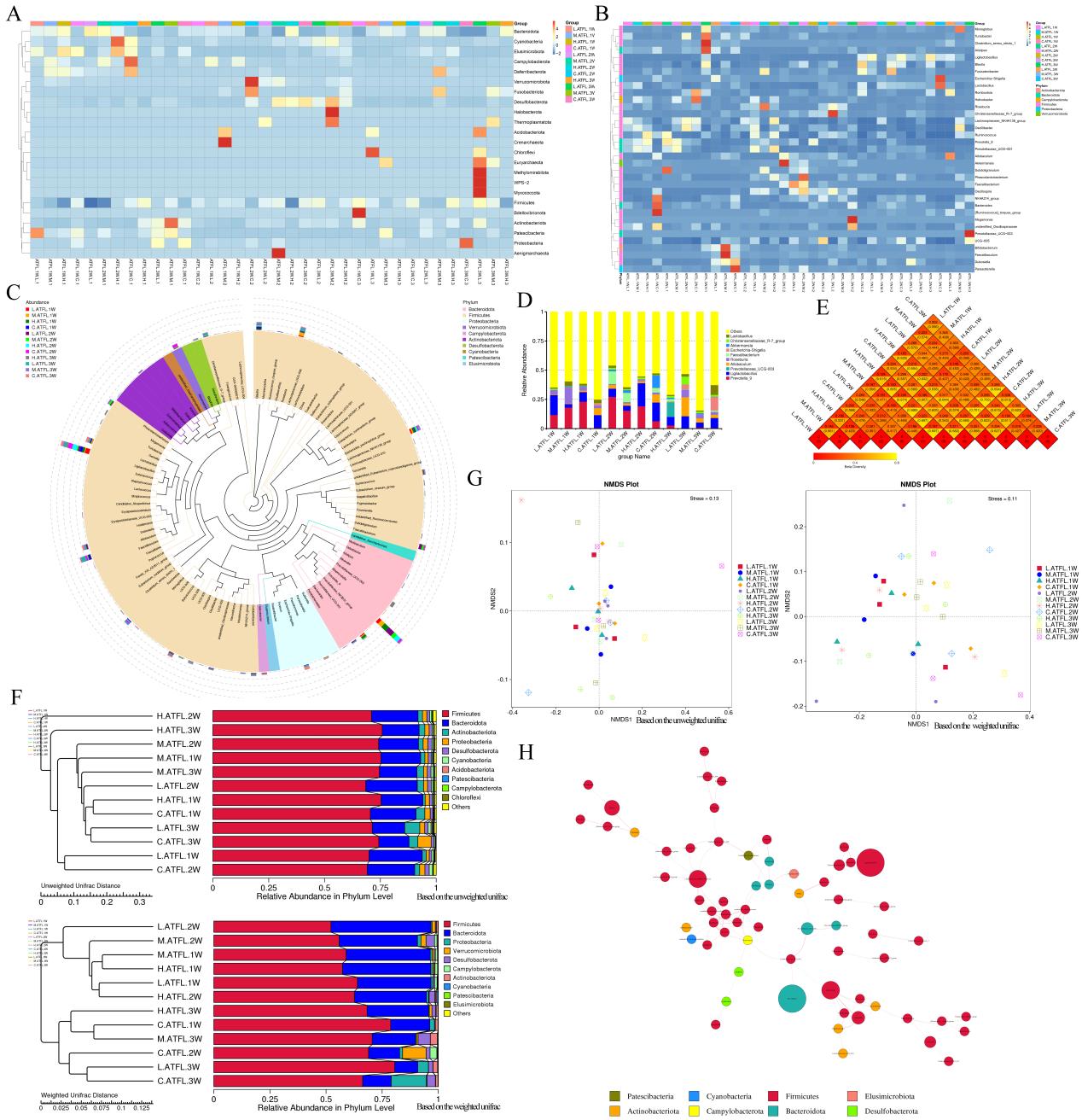
**

**Supplementary figure.** Changes in the intestinal OTUs of ATFL rats after TENS treatment

A. The species abundance clustering plot of samples at phylum level. B. The species abundance clustering plot of samples at genus level. The abscissa is the sample name; The ordinate represents the relative abundance. C. The representative sequences of the top 100 genus were analyzed by multiple sequence alignment. The colors of branches and sectors is the corresponding phyla, and the stacked bar plots on the outside of the fan ring is the abundance distribution of the genus. D. The top 10 genera of TENS groups. Others are the sum of the relative abundances of all other genera outside the top 10 genera. E. Beta diversity analysis of ATFL rats after TENS treatment. The square number is the dissimilarity coefficient between two samples. The smaller the dissimilarity coefficient, the smaller the difference of species diversity between two samples. In one square, the upper and lower values represent weighted unifrac and unweighted unifrac distance, respectively. F. The weighted and unweighted unifrac distances of samples after TENS treatment. G. The beta-diversities of all samples which analyzed by NMDS. H. The relationship of microbiota in each group after treatment of TENS. The nodes represented as genera; The node size represented as the connectivity of genera; The same color represented as the same gate level.
